# Highlighting the Proteasome: Using Fluorescence to Visualize Proteasome Activity and Distribution

**DOI:** 10.3389/fmolb.2019.00014

**Published:** 2019-03-22

**Authors:** Jin Gan, Yves Leestemaker, Aysegul Sapmaz, Huib Ovaa

**Affiliations:** Department of Cell and Chemical Biology, Oncode Institute, Leiden University Medical Centre, Leiden, Netherlands

**Keywords:** proteasome, fluorescence, activity, distribution, probe

## Abstract

Proteasomes are critical proteases in the cell responsible for the turnover of many cytoplasmic and nuclear proteins. They are essential for many cellular processes and various diseases are associated with their malfunctioning. Proteasome activity depends on the nature of the catalytic subunits, as well as the interaction with associated proteasome regulators. Here we describe various fluorescence-based methods to study proteasome function, highlighting the use of activity-based probes to study proteasome localization, dynamics, and activity in living cells.

## Introduction

To maintain cellular homeostasis, cells must balance the synthesis and degradation of cellular constituents. Proteins can fail to fold correctly during protein synthesis or can become damaged under various stress conditions e.g., oxidative stress. When this occurs, aberrant proteins must be swiftly removed to protect the cell from undesired protein activity and the formation of potentially harmful protein aggregates (Glickman and Ciechanover, 2002). In addition, the selective and timely removal of intracellular signaling proteins by degradation is important for the regulation of protein signaling pathways. In mammalian cells, degradation of intracellular proteins is carried out by two major pathways. The first pathway is the ubiquitin-proteasome system (UPS) and it is responsible for ~70% of all intracellular protein degradation in proliferating cultured cells (Rock et al., 1994). The second degradation pathway is autophagy which results in the lysosomal degradation of cellular components and organelles (Ebrahimi-Fakhari et al., 2011). The UPS unfolds and cleaves proteins into small polypeptide fragments with a typical length between 8 and 10 amino acids. These fragments can be broken down further by other proteases providing a source of amino acids for the synthesis of new peptides. In addition, these peptide fragments can be presented by the human major histocompatibility complex-1 (MHC-I), which is present on the cell surface of all nucleated cells, to cells of the immune system (Groettrup et al., 2010). Since protein degradation is essential for many cellular processes, disruption of normal proteasome function can contribute to disease (Glickman and Ciechanover, 2002). The proteasome has been implicated to play a role in neurodegenerative diseases, various cancers, immune-related diseases, and aging (Schmidt and Finley, 2014). Modulation of 26S proteasome activity has a proven therapeutic potential (Eldridge and O'Brien, 2010). Since the regulation of 26S proteasome activity is complex, there is a high demand for assay reagents that can report both proteasome activity as well as localization. This review provides a summary of the fluorescent tools that are currently available. Table 1 provides an overview of the reagents discussed below.

**Table 1:** An overview of reagents or techniques (typical examples) to study proteasome activity or localization.

**Classes**	**Examples**	**Activity**	**Localization**	**Cell permeability**	**References**
Peptide-based model substrates	β1: Z-LLE-AMC, Z-LLE-NA, Ac-nLPnLD-AMC, AC-GPLD-AMC, Z- nLPnLD-aminoluciferin	Yes	No	No	Kisselev and Goldberg, 2005; Moravec et al., 2009
	β2: Bz-VGR-AMC, Boc-LRR-AMC, Z-ARR-AMC, Bz-FVR-AMC, Boc-LSTR-AMC, Ac-RLR-AMC, Z-LRR-aminoluciferin	Yes	No	No	
	β5: Suc-LLVY-AMC, Z-GGL-AMC, Suc-AAF-AMC, Suc-LLVY-aminolufiferin	Yes	No	No	
	Nonapeptides: LFP, LF-2	Yes	No	No	Smith et al., 2005; Jastrab et al., 2015
	FRET reporter 1	Yes	No	No	Coleman and Trader, 2018
Protein-based model substrates	Ub_4_(K48)-Ub-GFP-Tail, Ub_8_(K48)-Ub-GFP-Tail, Ub_4_(K63)-Ub-GFP-Tail, Ub_4_(K11)-Ub-GFP-Tail, Tail-GFP-Ub-Ub_4_(K63), Ub_2_(K48)-Ub_2_(K48)–GFP-Tail	Yes	No	No	Martinez-Fonts and Matouschek, 2016
	Poly-ubiquitinated substrate (with polyubiquitin chains and Alexa Fluor 546 dye)	Yes	No	No	Bhattacharyya et al., 2016
	Ub_4_(lin)-GFP-Tail	Yes	No	No	Singh Gautam et al., 2018
	UbL^Rad23^-GFP-95	Yes	No	No	Yu et al., 2016
Fluorescently-tagged proteins	ODC-GFP, Ub-R-GFP, Ub-L-GFP	Yes	No	N/A	Li et al., 1998; Dantuma et al., 2000
	YFP-Plk1	Yes	No	N/A	Lindon and Pines, 2004
	GFP-β1i	No	Yes	N/A	Reits et al., 1997
	DQ-ovalbumin	Yes	Yes	No	Rockel et al., 2005
Deg-On system	Deg-On, eDeg-On	Yes	No	N/A	Zhao et al., 2014
Subunit specific ABPs^*^	β1c/ β1i- selective ABP	Yes	No	Yes	van Swieten et al., 2007
	β1i/β1c: Cy5-NC001, BodipyFL-NC001, BodipyFL-LU001c, Cy5-LU001i	Yes	No	No	de Bruin et al., 2016a,b
	β2i/β2c: BODIPY(FL)-LU112, Cy5-LU112	Yes	No	No	
	β5i/β5c: BODIPY(TMR)-NC005, Cy5-LU015, BodipyFL-LU015, BodipyFL-LU015c, Cy5-LU035i	Yes	No	No	
Pan-reactive ABPs^*^	Dansyl-Ahx3-L3-VS	Yes	Yes	Yes	Berkers et al., 2005
	BodipyFL-Ahx3-L3-VS	Yes	Yes	Yes	Berkers et al., 2007
	BodipyTMR-Ahx3-L3-VS	Yes	Yes	Yes	Verdoes et al., 2006

* *There are comprehensive summaries about these probes available in other review articles (Carmony and Kim, 2013; Hewings et al., 2017)*.

## Overview of Human Proteasome

The 26S proteasome is a multi-subunit, ATP-dependent protease complex. It consists of a 20S core particle (20S CP) capped on one or both sides by a 19S regulatory particle (19S RP) (Groll et al., 2000). The 20S CP is composed of four stacked rings, each consisting of seven subunits. The outer two rings contain seven similar, yet distinct alpha subunits (named α1–α7). The inner two rings of the 20S CP consist of seven distinct beta subunits (named β1–β7). Three of these beta subunits contain active sites with proteolytic activity. The constitutively expressed catalytically active subunits are β1, β2, and β5, which display caspase-like, trypsin-like, and chymotrypsin-like activity, respectively. The 19S RP is involved in the recognition, binding, deubiquitination, and unfolding of polyubiquitinated proteins. It is also involved in the translocation of the polypeptide chain into the interior of the 20S CP. Alternative regulatory particles have been reported, including the PA28αβ and PA28γ protein complexes, the PA200 proteasome-activating protein, and PI31 (Li et al., 2014).

In addition to the different regulatory particles, different isoforms of the 20S CP have also been described. In lymphoid tissues, or after stimulation with interferon γ (IFN-γ) in non-lymphoid tissues, the constitutive β subunits can be replaced by the immunoproteasome subunits β1i, β2i, and β5i to form the immunoproteasome. Mixed-type proteasomes containing a combination of constitutive and immunoproteasomes have been described (Dahlmann et al., 2000). Also, proteasomes expressing tissue-specific subunits such as the thymoproteasome (containing β5t instead of β5) (Murata et al., 2007) and the testis-specific proteasome (containing α4s instead of α4) have been observed (Uechi et al., 2014).

Proteasome activity is dynamically regulated depending on changing cellular needs. For instance, during fundamental cellular processes such as apoptosis, proliferation, or differentiation, the activity of the proteasome is altered (Schmidt and Finley, 2014). Environmental factors such as oxidative stress, disease states, or small molecules, can influence 26S proteasome activity as well (Aiken et al., 2011). One way in which proteasome activity is regulated is by post-translational modifications. Proteasomal subunits, like many other proteins, can be modified by phosphorylation (VerPlank and Goldberg, 2017), N-acetylation, alkylation, O-glycosylation, S-glutathionylation, N-myristoylation, and oxidation of sulfur-containing amino acid residues (Sorokin et al., 2009). These modifications affect both the activity as well as the localization of the 26S complex. Proteasomes have also been shown to interact with a growing list of proteasome-interacting proteins (Hartmann-Petersen and Gordon, 2004), including chaperones, E3 ligases, and deubiquitinases which may lead to altered stability of the 26S proteasome complex and/or its proteolytic activity (Tai et al., 2010).

Additionally, proteasome activity can be regulated by small molecule compounds (Huang and Chen, 2009). A wide variety of synthetic and natural inhibitors have been reported in the past 25 years. Proteasome inhibitors including bortezomib and carfilzomib have been used therapeutically for treatment of multiple myeloma and mantle cell lymphoma in patients (Kisselev et al., 2012; Teicher and Tomaszewski, 2015). In contrast, several drugs that increase 26S proteasome activity have potential applications in the treatment of neurodegenerative diseases (Myeku and Duff, 2018).

## Visualizing Proteasome Activity

### Peptide-Based Model Substrates

The activity of both 20S and 26S proteasomes can be measured using small peptide-based substrates. This is the most classical way to study proteasome activity. These substrates are typically three to four amino acids in length and are attached to a fluorescent reporter molecule such as 7-amino-4-methylcoumarin(AMC) (Kisselev and Goldberg, 2005) or a bioluminescent reporter molecule such as aminoluciferin (Moravec et al., 2009). After cleavage of the substrate by the proteasome, the reporter molecule is no longer caged. For AMC-based substrates, the fluorescent signal can be detected directly, while aminoluciferin needs to be processed further by luciferase to generate signal. For each of the different catalytic activities of the proteasome, there are specific peptide substrates available (Table 1). This allows the different catalytic activities of the proteasome to be measured separately. Unfortunately, many fluorogenic substrates are not cell-permeable, and therefore only applicable for studying purified proteasomes, permeabilized cells, or cell lysates. Alternatively, the substrate Suc-LLVY-AMC had been microinjected into cell nuclei to investigate proteasome activity in living cells (Rockel et al., 2005). Also, most substrates are processed by both the constitutive and immunoproteasome catalytic subunits of the 20S CP. Some substrates can be non-specifically processed by other proteases besides the proteasome leading to high levels of background signal. Another shortcoming of these reagents is that they do not require poly-ubiquitination or processing by the regulatory particles, although the regulatory particles enhance the degradation rate of fluorogenic substrates by inducing 20S CP gate opening. Fluorogenic substrates also have an advantage of easily being applied for high-throughput screening (HTS). For example, Suc-LLVY-AMC was used as a probe to screen for small molecule agonists of purified 20S proteasome activity, and two compounds MK-866 and AM-404 were ultimately identified as bona fide stimulators (Trader et al., 2017). To improve the existing fluorescent peptide-based substrates, a peptide-based FRET reporter has been developed (Coleman and Trader, 2018). Compared to the classical peptide-based substrate described above, this reagent has a larger size resulting in slower degradation and increased dynamic range, and is also four times higher in sensitivity making it suitable for HTS. Nonapeptides are another type of fluorescence-based proteasome substrates. Just as the peptide-based FRET reporter, the larger molecule results in slower degradation by 20S proteasome compared to shorter substrates. This feature makes them an ideal tool to study proteasome activators. For example, nonapeptide LFP is slowly degraded because its entry to 20S proteasomes is prevented by the N termini of the α subunits, but PAN can stimulate LFP degradation as it triggers gate opening (Smith et al., 2005, 2007). Another nonapeptide, LF-2, was used in a study that identified a new proteasomal cofactor in *Mycobacterium tuberculosis* (Jastrab et al., 2015).

### Protein-Based Model Substrates

Peptide-based model substrates differ from native poly-ubiquitinated proteasome substrates in that the latter requires recognition and processing by the proteasome regulatory particles before proteolysis. For studying the entire degradation process by the 26S proteasome, poly-ubiquitinated model substrates would be valuable research tools. A dye-labeled poly-ubiquitinated substrate was described as an assay reagent for 26S proteasome activity. The N-terminus was enzymatically attached to a polyubiquitin chain to induce proteasome degradation, and the C-terminus was conjugated with Alexa Fluor 546 dye for fluorescence anisotropy measurements (Bhattacharyya et al., 2016). Moreover, several GFP-based substrates with polyubiquitin chains of defined lengths and specific linkages were developed for proteasome degradation assays (Martinez-Fonts and Matouschek, 2016). Recently, the same group described the development of a new model substrate consisting of linear tetraubiquitin fused to GFP expressing a degradation initiation region, which is highly suitable for HTS (Singh Gautam et al., 2018). However, producing these polyubiquitination chains is quite laborious, another type of substrate with ubiquitin-like (UbL) domains, rather than polyubiquitination chains are also available. UbL domains can also be recognized by the proteasome bypassing the need for ubiquitination. One example of such a substrate is UbL^Rad23^-GFP-95 (Yu et al., 2016).

### Intracellular Model Substrates

The poly-ubiquitinated model substrates reported so far are not cell-permeable. This is unfortunate, as it prevents us from studying the degradation of defined poly-ubiquitinated model substrates in a native environment. In mammalian cells it is possible to use overexpressed model substrates to determine 26S proteasome activity. A common strategy is to fluorescently tag a substrate protein, and monitor its degradation, such as YFP-Plk1 (Lindon and Pines, 2004). Another example of such a model substrate is GFP fused to 37 amino acids of ornithine decarboxylase (ODC), a protein which is degraded in an ubiquitin-independent manner (Li et al., 1998; Pegg, 2006). Other examples of overexpressed GFP-based model substrates are fusion proteins that contain N-end rule and ubiquitin fusion degradation (UFD) signals such as ubiquitin-R-GFP and ubiquitin-L-GFP (Dantuma et al., 2000). The Deg-on system is an expression-based system that translates the level of 26S proteasome activity into a fluorescent output. In this system, the expression of GFP is repressed by a continuously expressed genetically encoded proteasome substrate. When proteasome activity is increased, the level of the proteasome substrate goes down, resulting in less GFP protein repression. This results in an increased level of GFP, which can be detected. Vice versa, when proteasome activity is decreased, the levels of the proteasome substrate will rise. This will increase GFP repression, leading to lower levels of GFP (Zhao et al., 2014).

### Activity-Based Proteasome Probes

**Figure 1:**
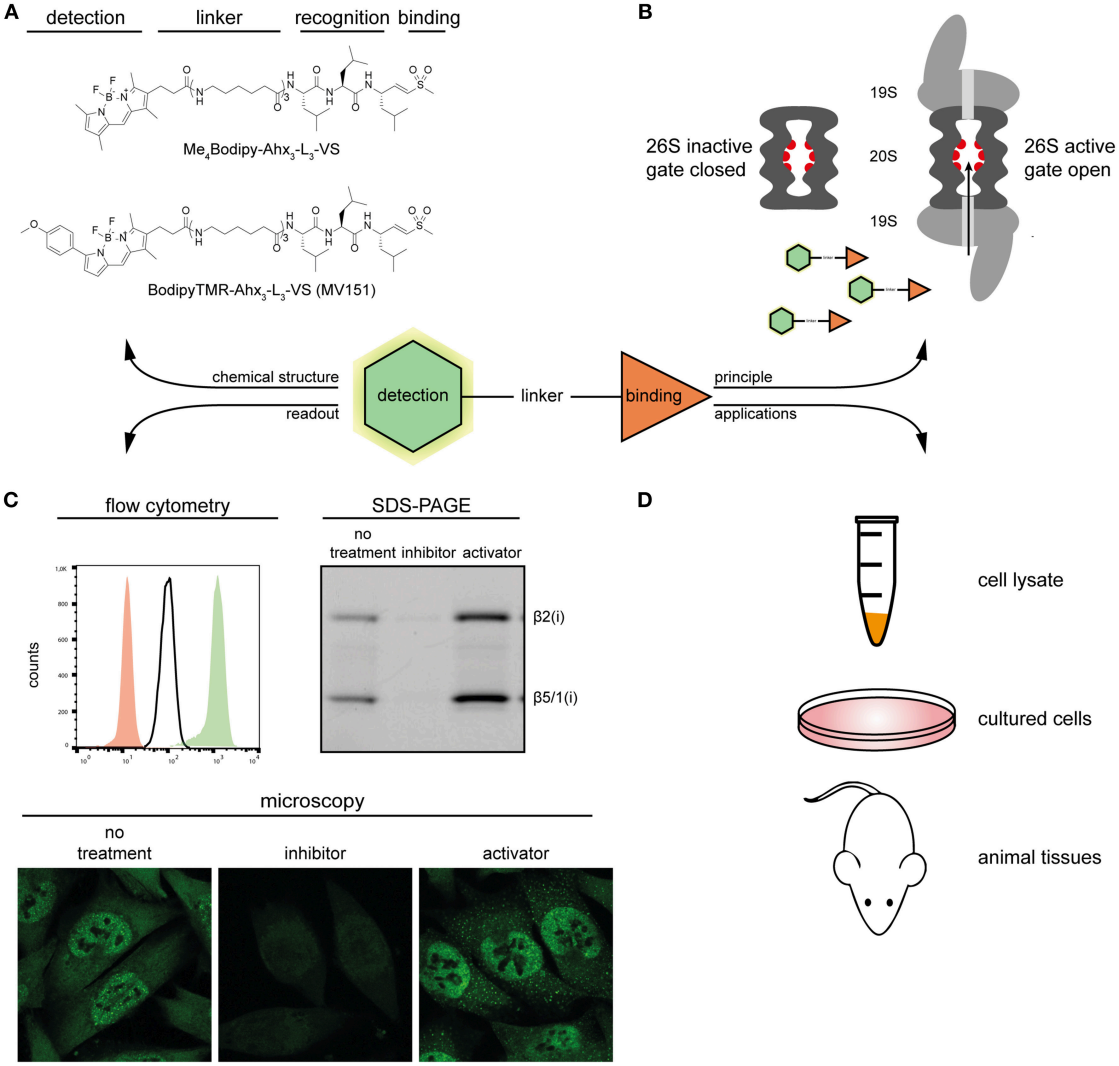
Overview of proteasome ABPs. **(A)** Molecular structures of two proteasome ABPs. **(B)** The principle of how probes target the active proteasome: proteasome ABPs enter through the 20S proteasome gate, and covalently target the catalytic sites. **(C)** Typical examples of the detection methods of proteasome ABPs. Left, overlay of the ABP signal in proteasome inhibitor treated (red), untreated (white), and proteasome activator treated (green) MelJuSo cells; Right, In-gel fluorescence scan showing representative proteasome activity profiles of proteasome inhibitor treated, untreated, and proteasome activator treated MelJuSo cells; Below, confocal microscopy images of the ABP signal in proteasome inhibitor treated, untreated and proteasome activator treated MelJuSo cells. **(D)** Applications of proteasome ABPs.

Activity-based proteasome probes (ABPs) are developed based on the covalent binding of small inhibitors with active site residues of catalytic subunits. A typical ABP consists of a warhead, a recognition element and a reporter tag (Figure 1A). The recognition element, either a small polypeptide, a small molecule, or a protein derivative, directs the probe to active enzyme for enhanced selectivity. Then the warhead with modest reactivity covalently reacts with the catalytic residues. The reporter tag can be an affinity tag such as biotin to allow for isolation or a fluorophore for fluorescence signal detection. The proteasome ABPs are generally classified as either subunit specific ABPs or broad spectrum ABPs based on their selectivity toward a specific or all of the catalytic subunits.

Broad spectrum ABPs are reactive to all proteasome catalytical subunits. These probes gain access to the binding target through the gated channel of the 20S core particle rather than random diffusion. If the gate is closed, or the binding sites are occupied by a proteasome inhibitor (e.g., MG132), the fluorescence signal will decrease. Conversely, if the gate is open, more probe can enter into the 20S core particle, and the fluorescent signal will increase (Figure 1B). Dansyl-Ahx_3_-L_3_-VS was the first reported cell-permeable and directly detectable broad spectrum ABP (Berkers et al., 2005). It was subsequently optimized into two other classical proteasome probes BodipyTMR-Ahx_3_-L_3_-VS (MV151) (Verdoes et al., 2006) and Me_4_Bodipy-Ahx_3_-L_3_-VS (Berkers et al., 2007), by replacing the dansyl group with Bodipy fluorophores. This change made the probe more sensitive for fluorescence detection while keeping its activity-based and cell-permeable properties. This means that these probes can be used in cell lysates, living cells, as well as animal tissues (Figure 1D), and are suitable for a variety of monitoring techniques, including in-gel fluorescence scan, flow cytometry, and fluorescence microscopy (Figure 1C). All these features make both of these probes widely used in proteasome-related studies nowadays.

The Me_4_Bodipy-Ahx_3_-L_3_-VS probe was used in a flow cytometry-based HTS. Eleven small molecule compounds were identified as novel proteasome activators in living cells, and the p38 MAPK pathway was highlighted as a novel signaling pathway to modulate proteasome activity (Leestemaker et al., 2017). In another study, thermal proteome profiling revealed that CDK4/6 inhibitor palbociclib induces stabilization of the 20S proteasome complex. When MCF7 cells were treated with palbociclib, the proteasome activity also increased as measured using Me4BodipyFL-Ahx_3_-L_3_-VS probe (Miettinen et al., 2018). Furthermore, the MV151 can also label the β2 and β5 subunits of the Plasmodium proteasome and was applied in a flow cytometry-based screen to identify Plasmodium specific inhibitors that selectively kill parasites (Li et al., 2012). Beyond applications in cultured cells, both probes are broadly used in animal tissues, providing an easier way to study changes in proteasome activity and drug bioavailability when mice are administrated with proteasome inhibitors and activators (Verdoes et al., 2006; Berkers et al., 2007). In a recent study, isolated neural stem cells treated with Me_4_BodipyFL-Ahx_3_-L_3_-VS probes revealed that quiescent neural stem cells (NSCs) have reduced proteasome activity compared to activated NSCs (Leeman et al., 2018). The MV151 probe has also been applied to plant science. When the Arabidopsis plants were sprayed with benzothiadiazole (BTH), the cytoplasmic proteasome activation could be monitored by MV151 using an in-gel fluorescence scanning approach (Gu et al., 2010).

Subunit-specific ABPs have a strong preference for a specific subunit type in an optimized range of probe concentration and reaction time. A comprehensive review article about these probes was recently published (Hewings et al., 2017). For example, a combination of subunit-specific ABPs with different fluorophores can enable visualization of all six catalytic subunits simultaneously by standard SDS-PAGE gel (de Bruin et al., 2016b). This combination was then used to identify new subunit selective compounds, such as β5c selective inhibitors (Xin et al., 2016). However, as these ABPs display poor cell permeability, efficient labeling requires the use of cell lysates. A cell-permeable β1c/ β1i- selective ABP is available, but the fluorescence labeling requires a two-step approach (van Swieten et al., 2007).

## Visualizing Proteasome Distribution And composition

### Proteasome Marker Antibodies

The canonical approach to visualize proteasome distribution and composition is to use commercially available antibodies targeting various proteasome subunits in combination with microscopy. This approach is broadly applicable for all types of cells and with fixed tissues. However, this technique is quite invasive, as cell fixation, and permeabilization are necessary for antibody staining. Therefore, the accuracy of proteasome complex localization might be affected under the harsh treatment, like fixation with cold methanol. In addition, antibodies cannot differentiate between active and inactive proteasomes.

### Fluorescently-Tagged Approach

Fluorescently-tagged proteasome subunits have been widely used to visualize proteasome distribution and dynamics in living cells for over two decades. GFP-β1i was the first subunit to be overexpressed and incorporated into the proteasome (Reits et al., 1997). Afterwards, several other subunits were also fluorescently-tagged and integrated into the proteasome (Salomons et al., 2010). The intracellular distribution of fluorescently-tagged proteasomes can be easily visualized in living cells under a fluorescence microscope (Enenkel, 2014). The dynamics of proteasomes can also be followed over time by photobleaching a small area in a living cell. Strategies include fluorescence recovery after photobleaching (FRAP) and fluorescence loss in photobleaching (FLIP) (Groothuis and Reits, 2005). Fluorescence correlation spectroscopy (FCS), was also used to study the concentration, dynamics, and complex formation of the 26S proteasome in living yeast cells (Pack et al., 2014).

In addition, fluorescently tagged substrates can be used to study proteasome distribution. For example, the fluorogenic protein DQ-ovalbumin (DQ-Ova) was microinjected into the nuclei of cultured human cells to study the distribution of proteasome degradation in different nuclear compartments (Rockel et al., 2005).

Furthermore, the co-localization or interaction between proteasome and substrate is observable by co-expressing differently tagged proteasome subunits and substrate proteins (Schipper-Krom et al., 2014). A shortcoming of this approach is that the fluorescence does not necessarily represent intracellular distribution of intact active proteasomes, because not all of the tagged subunits are efficiently incorporated in the proteasome complexes. The non-incorporated fractions can interfere with proteasome distribution. In addition, fluorescent pre-complexes without activity also exist in cells. Incorporation of subunits in proteasome complexes can be determined by several laborious ways: (1) proteasome complex immunoprecipitation with antibodies against different subunits; (2) sucrose density centrifugation or native gradient PAGE; (3) diffusion rate determination of the fluorescent subunits (Groothuis and Reits, 2005; Enenkel, 2012).

### ABP Based Approach

Proteasome ABPs target the active proteasome specifically and show a similar proteasome distribution pattern when compared to fluorescently-tagged proteasomes in living cells. The dynamics of active proteasomes in living cells can be observed when cells are pretreated with proteasome inhibitors (Berkers et al., 2007). When mice are administered with MV151, proteasome activity profiles in different organs or tissues can also be visualized (Verdoes et al., 2006). Besides visualizing proteasome distribution, proteasome ABPs are valuable tools in studying proteasome composition, especially subunit-specific ABPs. Two different studies described the development of a set of FRET donors and acceptors that selectively target the proteasome catalytic subunits (Park et al., 2014; de Bruin et al., 2016a). Such reagents can be used to determine the different proteasome subtypes present in cells, i.e., distinguishing constitutive proteasomes from immunoproteasomes and mixed-type proteasomes. However, as these reagents are not cell-permeable, their application is limited to studying purified proteasomes, or cell lysates.

## Concluding remarks

Substrate-based fluorescent reporters are useful tools for visualizing proteasome activity but cannot be used to study the intracellular localization of active proteasomes. While use of antibodies and fluorescently-tagged proteasome subunits are ideal approaches to visualize proteasome distribution and dynamics, they do not demonstrate proteasome activity in cells. The newly developed ABPs are valuable bi-functional reagents to studying both proteasome activity and distribution.

Proteasome ABPs offer a series of valuable advantages over traditional assays due to some inherent features. First, ABPs display the availability and reactivity of the active proteasomes, rather than abundance, while antibody-based approaches detect the active and inactive forms of proteasome indiscriminately. Second, ABPs are applicable for use with cell lysates or living cells, instead of being limited to assays using purified proteasomes. Third, the proteasome activity can be monitored without prior knowledge of a natural or artificial substrate of the proteasome, which remains a bottleneck for many other assays. However, there are still some limitations for proteasome ABPs, the labeling is a covalent and irreversible reaction between the target and the probe. Therefore, labeled proteins are no longer active, and this may affect the subsequent cellular pathways in living cells. Despite this, proteasome ABPs can play more important roles in proteasome related studies.

## Author Contributions

JG, YL, and HO: conception and design; JG and YL: manuscript writing; JG, YL, AS, and HO: figure design and editing; HO: supervision. All authors approved the final version of the manuscript for publication.

### Conflict of Interest Statement

HO is a founder and shareholder of Ubiq Bio B.V. The remaining authors declare that the research was conducted in the absence of any commercial or financial relationships that could be construed as a potential conflict of interest.
